# Mitochondrial Ca^2+^ uniporter (MCU)-dependent and MCU-independent Ca^2+^ channels coexist in the inner mitochondrial membrane

**DOI:** 10.1007/s00424-013-1383-0

**Published:** 2013-10-27

**Authors:** Alexander I. Bondarenko, Claire Jean-Quartier, Warisara Parichatikanond, Muhammad Rizwan Alam, Markus Waldeck-Weiermair, Roland Malli, Wolfgang F. Graier

**Affiliations:** Institute of Molecular Biology and Biochemistry, Center of Molecular Medicine, Medical University of Graz, Harrachgasse 21/III, 8010 Graz, Austria

**Keywords:** Mitochondrial Ca^2+^ channels, Mitochondrial Ca^2+^ uniporter, MCU, Ca^2+^ signaling

## Abstract

A protein referred to as CCDC109A and then renamed to mitochondrial calcium uniporter (MCU) has recently been shown to accomplish mitochondrial Ca^2+^ uptake in different cell types. In this study, we investigated whole-mitoplast inward cation currents and single Ca^2+^ channel activities in mitoplasts prepared from stable MCU knockdown HeLa cells using the patch-clamp technique. In whole-mitoplast configuration, diminution of MCU considerably reduced inward Ca^2+^ and Na^+^ currents. This was accompanied by a decrease in occurrence of single channel activity of the intermediate conductance mitochondrial Ca^2+^ current (*i*-MCC). However, ablation of MCU yielded a compensatory 2.3-fold elevation in the occurrence of the extra large conductance mitochondrial Ca^2+^ current (*xl*-MCC), while the occurrence of bursting currents (*b-*MCC) remained unaltered. These data reveal *i-*MCC as MCU-dependent current while *xl-*MCC and *b*-MCC seem to be rather MCU-independent, thus, pointing to the engagement of at least two molecularly distinct mitochondrial Ca^2+^ channels.

## Introduction

Ca^2+^ uptake by mitochondria stimulates metabolic processes and can also initiate cell death pathways (for review, see [5, 8]). Accordingly, mitochondrial Ca^2+^ channels represent promising molecular targets for future therapeutic modulation of mitochondria functions. A precise understanding of the molecular mechanisms of mitochondrial Ca^2+^ uptake, molecular structure, and function of mitochondrial Ca^2+^ channels is required. Therefore, identification and electrophysiological characterization of mitochondrial Ca^2+^ channels and especially pinpointing specific channel activity to specific proteins will provide invaluable insight into actual processes that accomplish mitochondrial Ca^2+^ uptake.

Although several proteins have been identified to contribute to mitochondrial Ca^2+^ uptake, such like the mitochondrial Ca^2+^ uptake 1 (MICU1) [18], uncoupling proteins 2 and 3 [22, 23], ryanodine receptors [20, 21], mitochondrial Ca^2+^ uniporter regulator 1 (MCUR1) [14], and the canonical transient receptor potential 3 channel [6], the mitochondrial Ca^2+^ uniporter, MCU, a transmembrane protein in the inner mitochondrial membrane, has been proposed to be dominantly responsible for mitochondrial Ca^2+^ uptake [1, 4]. Recent advancement of the patch-clamp approach using mitoplasts allowed to identify mitochondrial Ca^2+^ uniport as a highly Ca^2+^-selective ion channel [13] that was dependent on the presence of MCU [3]. Moreover, MCU-established currents were sensitive to ruthenium red, which has been assumed to be a classic feature of the mitochondrial Ca^2+^ uniport. A point mutation in the putative pore domain of MCU decreased the sensitivity of the respective Ca^2+^ current to ruthenium red without changing the current magnitude [3]. However, integral Ca^2+^ currents through whole mitoplasts presented in the study of Chaudhuri et al. do not enable to discriminate between contributions of different single channel conductances [3]. Single channel recordings allowed to characterize more than one ruthenium red-sensitive Ca^2+^ inward current in mitoplasts isolated from cardiac myocytes (mitochondrial Ca^2+^ currents 1 and 2; mCa1, mCa2) [17], mitochondrial ryanodine receptor channel activity [21], endothelial cells [small mitochondrial Ca^2+^ currents, intermediate mitochondrial Ca^2+^ currents (*i-*MCC), and large mitochondrial Ca^2+^ currents (*l-*MCC)] [9], and HeLa cells [*i-*MCC and the extra large mitochondrial Ca^2+^ current (*xl-*MCC)] [2, 9], thus challenging the concept of MCU being the one and only Ca^2+^ channel in the inner mitochondrial membrane. The present study was designed to characterize the impact of MCU knockdown on different Ca^2+^ currents in mitoplasts isolated from HeLa cells by applying electrophysiological recordings in whole-mitoplast and mitoplast-attached configurations. These experiments were complemented with fluorescent mitochondrial Ca^2+^ measurements in the respective wild type and MCU knockdown (MCU-KD) HeLa cells. We show that in divalent-free conditions, Na^+^ readily permeates ruthenium red (RuR)-sensitive Ca^2+^ channels and downregulation of MCU protein results in suppression of whole-mitoplast inward Na^+^ and Ca^2+^ currents and a decreased occurrence probability of *i*-MCC that was associated with a partial increase in occurrence of the *xl*-MCC [2].

## Materials and methods

### Design and production of stably MCU knockdown HeLa cells and their corresponding control cells

HeLa MCU-KD and HeLa control cells have been produced upon request and supplied by TeBu-bio® (Tebu-bio SAS, Le Perray-en-Yvelines Cedex, France). HeLa cells with stable MCU knockdown and the respective scrambled control cells were produced by applying the SilenciX® technology (Tebu-bio, www.tebu-bio.com, Le Perray-en-Yvelines, France) using the following 5′-3′shRNA sequence against MCU: GGTGCAATTTATCTTTATA.

### Cell culture and isolation of mitochondria

All cells were grown on DMEM containing 10 % FCS, 50 U/ml penicillin, and 50 μg/ml streptomycin. Mitochondria were freshly isolated as previously described [2]. Mitochondria were prepared from HeLa cells by differential centrifugation. Cells were trypsinized, harvested, and washed with PBS. The cell pellet was suspended in a 200-mM sucrose buffer containing 10 mM Tris-MOPS, 1 mM EGTA and protease inhibitor (1:50, P8340 Sigma, Vienna, Austria) (pH adjusted to 7.4 with TRIS), and homogenized with a glass–Teflon potter (40–50 strokes). Nuclear remnants and cell debris were centrifuged down at 900 g for 10 min. The supernatant was centrifuged at 3,000*g* for 20 min. The mitochondrial pellet was washed and centrifuged down at 7,000*g* for 15 min. All fractions were kept on ice until further utilization.

### Preparation of mitoplasts

Isolation and preparation of mitoplasts (mitochondria devoid of outer membrane) from HeLa cells was performed as recently described [2]. Briefly, mitoplast formation was achieved by incubation of isolated mitochondria in hypotonic solution (5 mM HEPES, 5 mM sucrose, 1 mM EGTA, pH adjusted to 7.4 with KOH) for 8 min. Then, hypertonic solution (750 mM KCl, 80 mM HEPES, 1 mM EGTA, pH adjusted to 7.4 with KOH) was added to restore isotonicity.

### Mitoplast patch-clamp recordings

Single channel measurements were performed in the mitoplast-attached configuration as previously described [2, 9]. In brief, patch pipettes were pulled from glass capillaries using a Narishige puller (Narishige Co., Ltd., Tokyo, Japan), fire-polished, and had a resistance of 8–12 MΩ. Mitoplasts were bathed in the solution containing (in millimolars): 145 KCl, 1 EGTA, HEPES, and pH adjusted to 7.2 with KOH. For single channel recordings, the pipette solution contained 105 mM CaCl_2_ and 10 mM HEPES, 10 μM cyclosporin A (Tocris Bioscience, Bristol, UK) and 10 μM 7-chloro-5-(2-chlorophenyl)-1,5-dihydro-4,1-benzothiazepin-2(3*H*)-one (CGP 37157, Ascent Scientific Ltd., Bristol, UK) to prevent opening of the permeability transition pore, and the activity of the mitochondrial Na^+^/Ca^2+^ exchanger (NCX_mito_), respectively. pH was adjusted to 7.2 with Ca(OH)_2_. Single channel currents were recorded at a fixed holding potential indicated in the respective figures. For whole-mitoplast recordings, pipette solution contained (in millimolars): 120 Cs methanesulfonate, 30 CsCl, 1 EGTA, 110 sucrose, 2 gluconic acid, and pH by TEAOH to 7.2. For obtaining whole-mitoplast configuration, voltage steps of 300–600 mV and 20–50 ms duration were applied. Voltage ramps of 1 s duration from −160 to +50 mV were delivered every 5 or 10 s from the holding potential 0 mV. Currents were recorded using a patch-clamp amplifier (EPC7, List Electronics, Darmstadt, Germany). Data collection was performed using Clampex software of pClamp (V9.0, Molecular Devices, Sunnyvale, CA, USA). Signals obtained were low pass filtered at 1 kHz using an eight-pole Bessel filter (Frequency Devices) and digitized with a sample rate of 10 kHz using a Digidata 1200A A/D converter (Molecular Devices, Sunnyvale, CA, USA). All measurements were performed at room temperature. For recording cationic currents via whole mitoplasts, bath solution contained (in millimolars): 150 TRIS HCl, 1 EGTA, 1 EDTA, 10 HEPES with pH 7.2. For I_Na_ recording, NaCl was substituted for TRIS HCl. Ca^2+^-containing bath solution for I_Ca_ recording contained (in millimolars): 140 TRIS HCl, 3 CaCl_2_, 10 HEPES, and pH 7.2

### Single cell Ca^2+^ imaging and data acquisition

Imaging mitochondrial targeted cameleon 4mtD3cpv was performed on a digital wide field imaging system, the Till iMIC (Till Photonics Graefelfing, Germany) using a 40× objective (alpha Plan Fluar 40×, Zeiss, Göttingen, Germany). For illumination of the cameleon, an ultrafast switching monochromator, the Polychrome V (Till Photonics), was used for excitation light at 430 nm. Emission light was collected at 480 and 535 nm using a single beam splitter design (Dichrotome, Till Photonics). Images were recorded with a charged-coupled device camera (AVT Stringray F145B, Allied Vision Technologies, Stadtroda, Germany). For the data acquisition and the control of the digital fluorescence microscope, the live acquisition software version 2.0.0.12 (Till Photonics) was used. Experiments were performed at the same day than the isolation, purification, and electrophysiological measurements of the respective mitoplasts.

### Experimental buffers for Ca^2+^ measurements

Ca^2+^ measurements in HeLa cells were performed by stimulating cells in Ca^2+^-containing environment. Cells were superfused by a Ca^2+^-containing buffer, which was composed of (in millimolars): 138 NaCl, 5 KCl, 2 CaCl2, 1 MgCl2, 10 d-glucose and 10 HEPES, and pH adjusted to 7.4 with NaOH. Stimulation was performed using 100 μM of the IP_3_-generating agonist histamine.

### Western blot

HeLa cells that were washed with ice-cold PBS or isolated mitochondria were lysed with RIPA buffer containing protease inhibitor cocktail (Sigma-Aldrich, Vienna, Austria). The protein concentration was measured using the BCA protein assay (Thermo Fisher Scientific Inc., Vienna, Austria). Forty micrograms of protein were separated by SDS-PAGE and transferred to a nitrocellulose membrane. The membrane was incubated with the primary antibody at 4 °C overnight and the primary antigen–antibody complex was detected by incubating the blot with a horseradish peroxidase-conjugated secondary antibody at room temperature for 2 h. The membrane was further developed with the ECL Plus Western blotting detection system (GE Healthcare, Vienna, Austria). To control the equal amount of protein loading of whole cell lysates and isolated mitochondria, MCU expression (sc-246071; Santa Cruz, Vienna, Austria) were densitometrically normalized to β-actin (sc-47778; Santa Cruz) and VDAC (sc-32063 and sc-32059; Santa Cruz), respectively.

### Real-time PCR

RNA was isolated from HeLa cells using a Total RNA isolation kit (PEQLAB Biotechnologie GmbH, Erlangen, Germany), and it was reverse transcribed using a High Capacity cDNA Reverse Transcription Kit (Applied Biosystems, USA). The analysis of the expression of the target genes was performed by conventional polymerase chain reaction (PCR) using GoTaq Green master mix (Promega, Madison, WI, USA) and real-time PCR using QuantiFast SYBR Green RT-PCR kit (Qiagen, Hilden, Germany) on LightCycler 480 (Roche Diagnostics, Vienna, Austria). RNA polymerase II (RPOL2) was used as a housekeeping control [12, 16, 24]. Primers for RPOL2 and MCU were obtained from Invitrogen (Vienna, Austria) and their sequences (5′–3′) were as follows: RPOL2: CATTGACTTGCGTTTCCACC, RPOL2 rev: ACATTTTGTGCAGAGTTGGC, MCU: TTCCTGGCAGAATTTGGGAG, and MCU rev: AGAGATAGGCTTGAGTGTGAAC.

### Statistical analysis

The occurrence probability was calculated as a fraction of patches displayed specific channel activity relative either to the total number of patches studied or the number of active patches displayed any type of the channel activity. Single channel analysis was performed using Clampfit 9.2 (Molecular Devices, Sunnyvale, CA, USA). Data are expressed as means with standard error. Statistical comparisons were conducted with a two-tailed unpaired *t* test. Values of *p* < 0.05 (*) were taken as statistically significant. Statistical analysis was performed by Graph Pad Software version 5.01 (La Jolla, CA, USA).

## Results

### Stably knockdown of MCU strongly reduced mitochondrial Ca^2+^ sequestration in intact HeLa cells

**Fig. 1:**
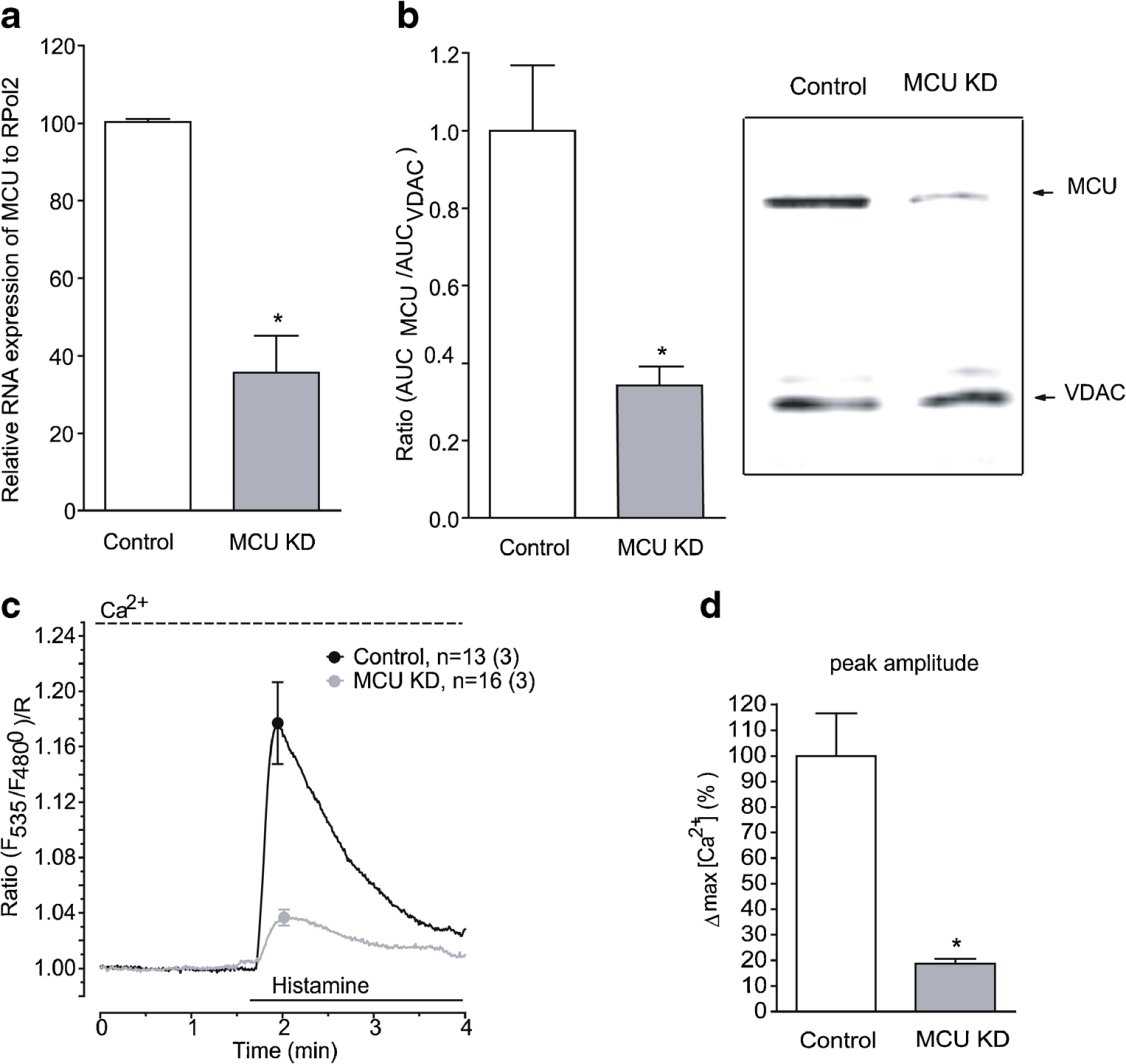
MCU knockdown impairs intramitochondrial Ca^2+^ rise during cell stimulation. **a** Relative RNA expression of control and MCU-KD cells, **b** MCU protein expression in control and MCU-KD cells. Representative bands for MCU and VDAC protein expression. **c** Averaged traces of mitochondrial Ca^2+^ signals upon stimulation with 100 μM histamine of intact control HeLa cell (*black trace*, *n* = 13 cells from three coverslips) and MCU-KD HeLa cells (*gray trace*, *n* = 16 cells from three coverslips). Mitochondrial Ca^2+^ signals were measured using cells expressing 4mtD3cpv. **d** Quantitative expression of intramitochondrial Ca^2+^ rise during cell stimulation with 100 μM histamine in MCU-KD cells relative to the rise in control cells

Diminution in MCU gene expression by stably expression of the respective shRNA in HeLa cells (MCU-KD cells) was confirmed by quantitative real-time PCR. In MCU-KD cells, the level of MCU mRNA expression was significantly depressed and amounted 36 ± 10 % (*n* = 3, *p* < 0.005) of the level detected in control cells (Fig.1a). Hence, Western blot analysis revealed that the cellular MCU protein content was attenuated to 33 ± 6 % (*n* = 2, *p* < 0.08) of the level detected in control cells (Fig.1b). In line with these findings, in MCU-KD cells, histamine-induced mitochondrial Ca^2+^ elevation was reduced to 19 % of the level attained in control cells (Fig.1c, d).

### Knockdown of MCU strongly reduced whole-mitoplast Ca^+^ currents

**Fig. 2:**
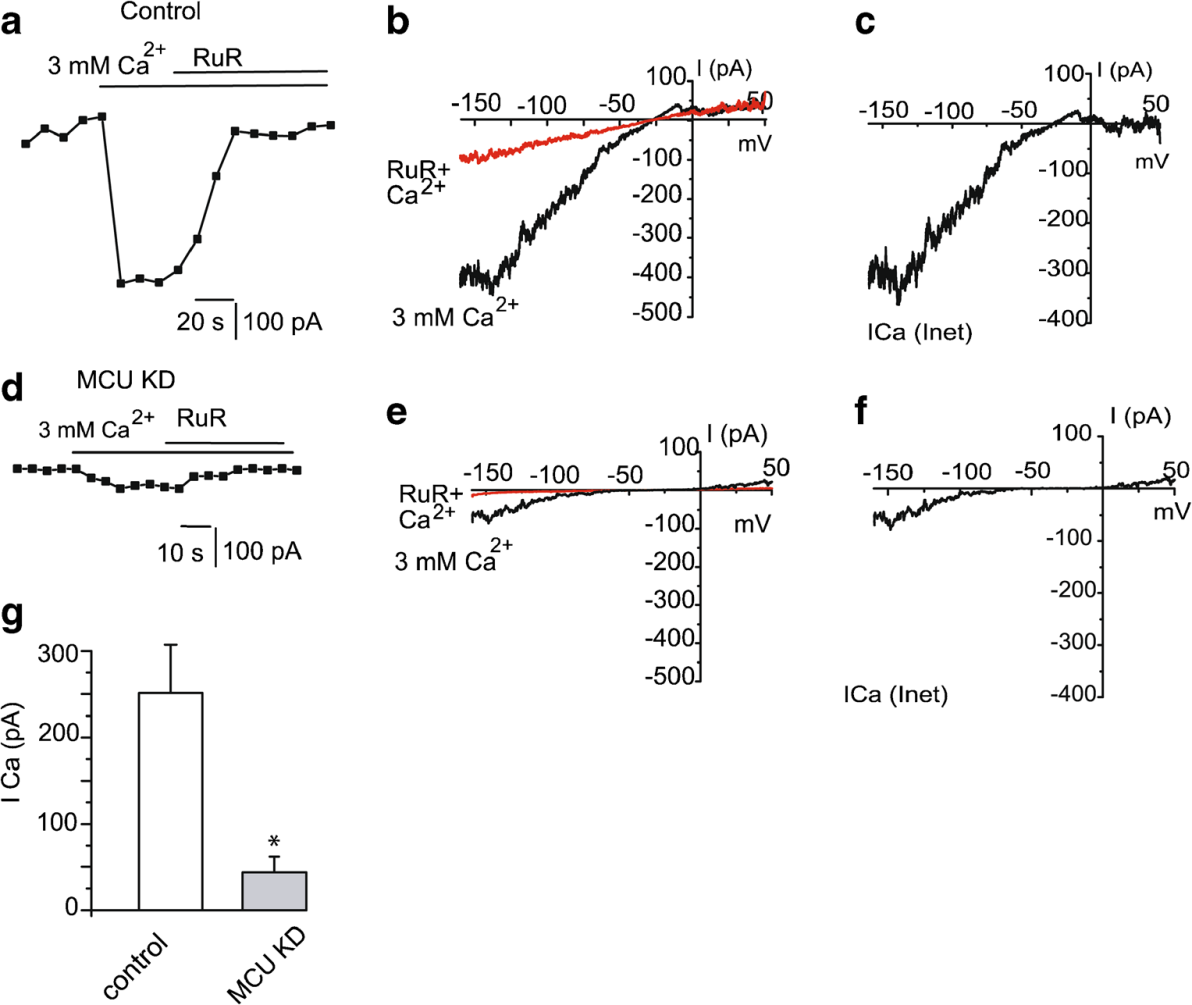
MCU knockdown suppresses whole-mitoplast Ca^2+^ current. **a** Exemplary time course of the whole-mitoplast current at −155 mV before and after addition of 3 mM Ca^2+^ followed by addition of 10 μM RuR (*n* = 4). Recording from mitoplast isolated from control cells. Voltage ramps were applied every 10 s. **b** Corresponding Ca^2+^ current responses to voltage ramps before and after addition of 10 μM RuR in the presence of 3 mM Ca^2^. **c** Net I_Ca_ obtained after subtraction of RuR-insensitive current. **d** Representative time course of whole-mitoplast currents at −155 mV induced by addition of 3 mM Ca^2+^ to the bath followed by addition of 10 μM RuR. Recoding from mitoplast isolated from MCU-KD cells. Voltage ramps were applied every 5 s. **e** Corresponding Ca^2+^ current responses to voltage ramps before and after addition of 10 μM RuR in the presence of 3 mM Ca^2+^ (*n* = 3). **f** Net I_Ca_ obtained after subtraction of RuR-insensitive current. **g** Mean amplitudes of mitochondrial C_a_ from control (*n* = 11) and MCU-KD (*n* = 5) mitoplasts

In whole-mitoplast configuration, switching from Ca^2+^-free to Ca^2+^-containing (3 mM) solution during voltage ramps from −160 to +50 mV resulted in an inward current at negative potentials (Fig.2a) that was sensitive to RuR (Fig.2b, c). In mitochondria isolated from MCU-KD cells, the current elicited by Ca^2+^ addition was strongly reduced (Fig.2d) to 17 % of the level attained in control cells, while it remained sensitive to RuR (Fig.2e, f). In control group, at −155 mV, the Ca^2+^ current amplitude averaged −251.4 ± 55.8 pA (*n* = 11), while in mitoplasts isolated from MCU-KD cells, the current averaged −43.5 ± 18.4 pA (*n* = 5) (Fig.2g). These results are very similar to that published very recently by the group of Clapham [3] and demonstrate that diminution of MCU results in a pronounced suppression of RuR-sensitive transmitochondrial inward I_Ca_ accompanied by potent reduction of intramitochondrial Ca^2+^ rise in intact cells exposed to supramaximal concentrations of histamine (Fig.1c).

### Diminution of MCU strongly reduced whole-mitoplast Na^+^ currents in the absence of Ca^2+^

**Fig. 3:**
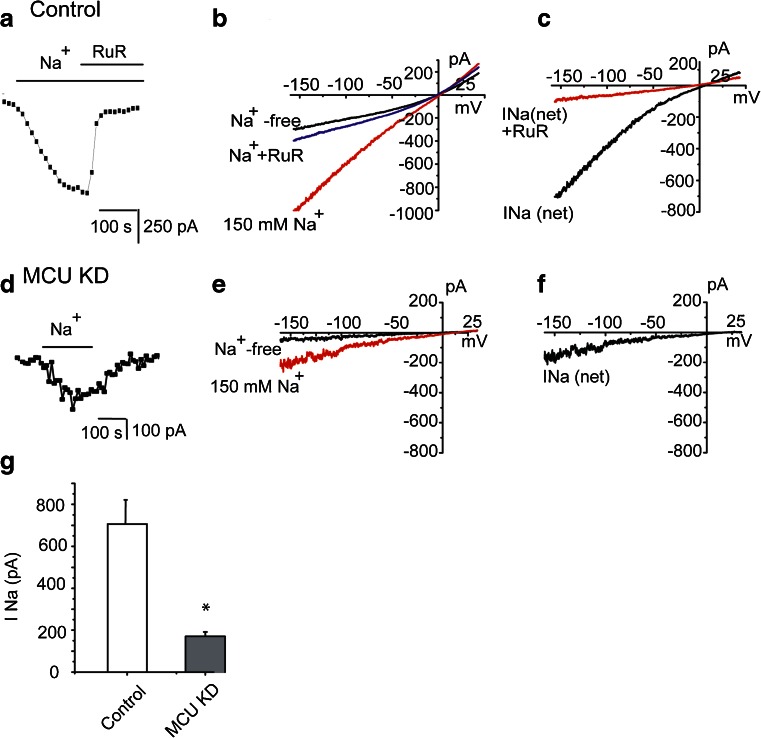
MCU knockdown suppresses whole-mitoplast Na^+^ current. **a** Exemplary time course of the whole-mitoplast current recorded from mitoplast isolated from control cells (*n* = 8) at −155 mV. The current was elicited by replacement of bath TRIS for Na^+^ in divalent-free conditions and was measured during voltage ramps applied every 10 s. **b** Corresponding current responses to voltage ramps before (Na^+^ free) and after addition of 150 mM Na^+^ either alone or in the presence of 10 μM RuR. **c** Net I_Na(Control)_ obtained after subtraction of the background current obtained in Na^+^ and divalent-free solution. **d** Representative time course of the whole-mitoplast current recorded from the mitoplast isolated from MCU-KD cells (*n* = 8) and measured at −155 mV during voltage ramps applied every 10 s before and after replacement of bath TRIS for Na^+^ in divalent-free solution (*n* = 8). **e** Corresponding current responses to voltage ramps before (Na^+^ free) and after application of 150 mM Na^+^ to MCU-KD mitoplast. **f** Net I_Na (MCU-KD)_ obtained after subtraction of background current. **g** Mean amplitudes of mitochondrial I_Na_ from control and MCU-KD groups

In whole-mitoplast configuration, switching from Na^+^-free to Na^+^-containing divalent-free solution resulted in a development of a pronounced inward current at negative potentials (Fig.3a). The current amplitude was a function of the applied membrane voltage. A mean current amplitude at −155 mV averaged 706 ± 142 pA (*n* = 8). The current rapidly terminated upon removal of bath Na^+^ and was inhibited by 10 μM RuR (Fig.3a–c). These observations indicate that in the absence of Ca^2+^, external Na^+^ readily permeates the RuR-sensitive channel(s), and the amplitude of transmitochondrial Na^+^ current (I_Na_) is higher than that generated by Ca^2+^ influx, an observation that is in line with previous studies [7, 13].

Stable knockdown of MCU resulted in a marked suppression of whole-mitoplast inward Na^+^ current (I_Na_) elicited by voltage ramps to 170.4 ± 21.0 pA (*n* = 8) that equals a reduction by 76 % (Fig.3d–g). These results demonstrate that MCU downregulation results in a pronounced suppression of transmitochondrial inward RuR-sensitive I_Na_.

### Stable knockdown of MCU reduces the occurrence of active single channels per patch

We next characterized the probability of occurrence of any single channel activities of mitochondrial Ca^2+^ channels in the mitoplast-attached configuration [2] under conditions of MCU knockdown. Among 67 patches tested in mitochondrial from MCU-KD cells, only 35 patches displayed single channel activity, providing 52 % occurrence. In mitoplasts isolated from control cells, single channel activity was detected more frequently in 71 out of 103 patches tested, providing an occurrence probability of 69 %. For statistical processing, we analyzed the occurrence probability of the channel activity for each individual experimental day and calculated the mean values and statistics out of the individual values from all experimental days (*N* ^D^). In the control group, the occurrence probability of single channel activities amounted 71 ± 6 % (*N* ^D^ = 32), while in MCU-KD group, the occurrence probability of single channel activities was significantly (*p* < 0.05) less and averaged 47 ± 8.0 % (*N* ^D^ = 13). Similar to mitoplasts from control group, in mitoplasts isolated from MCU-KD cells, we observed all three types of the channel activities described by us earlier [2].

### Stable knockdown of MCU reduces the occurrence of *i-*MCC

We next analyzed the proportion of each individual channel activity in the total number of patches tested and to the number of active patches, which would give an indication on the density of individual channel type in the overall population of Ca^2+^ channels.

**Fig. 4:**
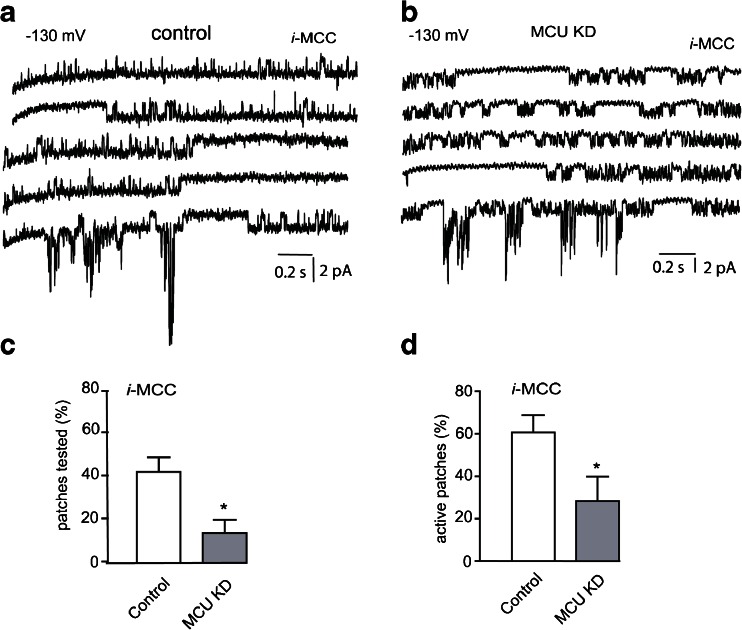
The occurrence probability of *i*-MCC channel is largely decreased in the inner mitochondria membrane from MCU-KD HeLa cells. **a** Representative single channel recording of *i*-MCC activity interrupted with *b*-MCC at a holding voltage of −130 mV in mitoplast isolated from control cells. **b** Representative recording of *i*-MCC interrupted with *b*-MCC at a holding voltage of −130 mV in mitoplast isolated from MCU-KD cells. **c** *Bars* represent the occurrence of *i*-MCC activity in mitoplasts from control and MCU-KD HeLa cells in respect to the total number of patches tested. **d** The same as in **c** but in respect to the number of active patches with any MCC activity

In mitochondria isolated from control cells, the most predominant channel was the 11 pS channel (intermediate conductance mitoplast Ca^2+^ channel, *i-*MCC) [2]. Exemplary traces of this type of activity in control and MCU-KD mitoplasts occasionally interrupted with bursting activity are depicted in Fig. 4a, b, respectively. Under control conditions, *i*-MCC activity was observed in 43 out of 103 patches tested (occurrence probability 42 ± 7 %, *N* ^D^ = 29) (Fig.4c) and its individual contribution to active channels (*n* = 71) was 61 ± 8 % (Fig.4d). In mitochondria from MCU-KD cells, the occurrence probability of this channel was strongly reduced compared to controls: the *i*-MCC channel activity was detected in 11 out of 67 patches tested (occurrence probability 14 ± 6 %, *N* ^D^ = 11) (Fig.4c) and its individual contribution to active channels (*n* = 35) was 28 ± 12 % (Fig.4d). In MCU-KD group, the *i*-MCC conductance (11.5 ± 0.5 pS, *n* = 6) was not different from that observed in mitoplasts isolated from control cells (11.9 ± 0.6 pS, *n* = 15). Gating characteristics of *i*-MCC were slightly affected by MCU knockdown and revealed a tendency for reduced open probability (NPo) and mean open time (To_mean_) while mean closed time (Tc_mean_) was prolonged (Table 1).

**Table 1:** The effect of MCU knockdown on gating characteristics of mitochondrial Ca^2+^ currents (*i*-MCC and *xl*-MCC)

	Conductance (pS)	NPo	To_mean_ (ms)	Tc_mean_ (ms)	*n*
*i-*MCC control	11.9 ± 0.5	0.60 ± 0.13	3.4 ± 0.5	14.2 ± 2.1	15
*i-*MCC MCU-KD	11.5 ± 0.6	0.36 ± 0.05	4.6 ± 0.8	17.3 ± 2.3	6
*p* value	0.70	0.26	0.17	0.42	
*b*-MCC control	25.7 ± 1.3	0.39 ± 0.08	2.7 ± 0.4	27.7 ± 4.9	8
*b*-MCC MCU-KD	26.0 ± 1.8	0.40 ± 0.13	1.6 ± 0.3*	12.5 ± 2.6*	9
*p* value	0.89	0.98	0.04	0.01	
*xl-*MCC control	74.8 ± 7.9	0.74 ± 0.08	31.2 ± 5.0	50.0 ± 14.4	6
*xl-*MCC MCU-KD	70.7 ± 5.9	0.40 ± 0.14*	12.1 ± 1.6*	44.7 ± 16.1	6
*p* value	0.690	0.002	0.006	0.248	

### Stable knockdown of MCU had no effect on the occurrence of the *b-*MCC

**Fig. 5:**
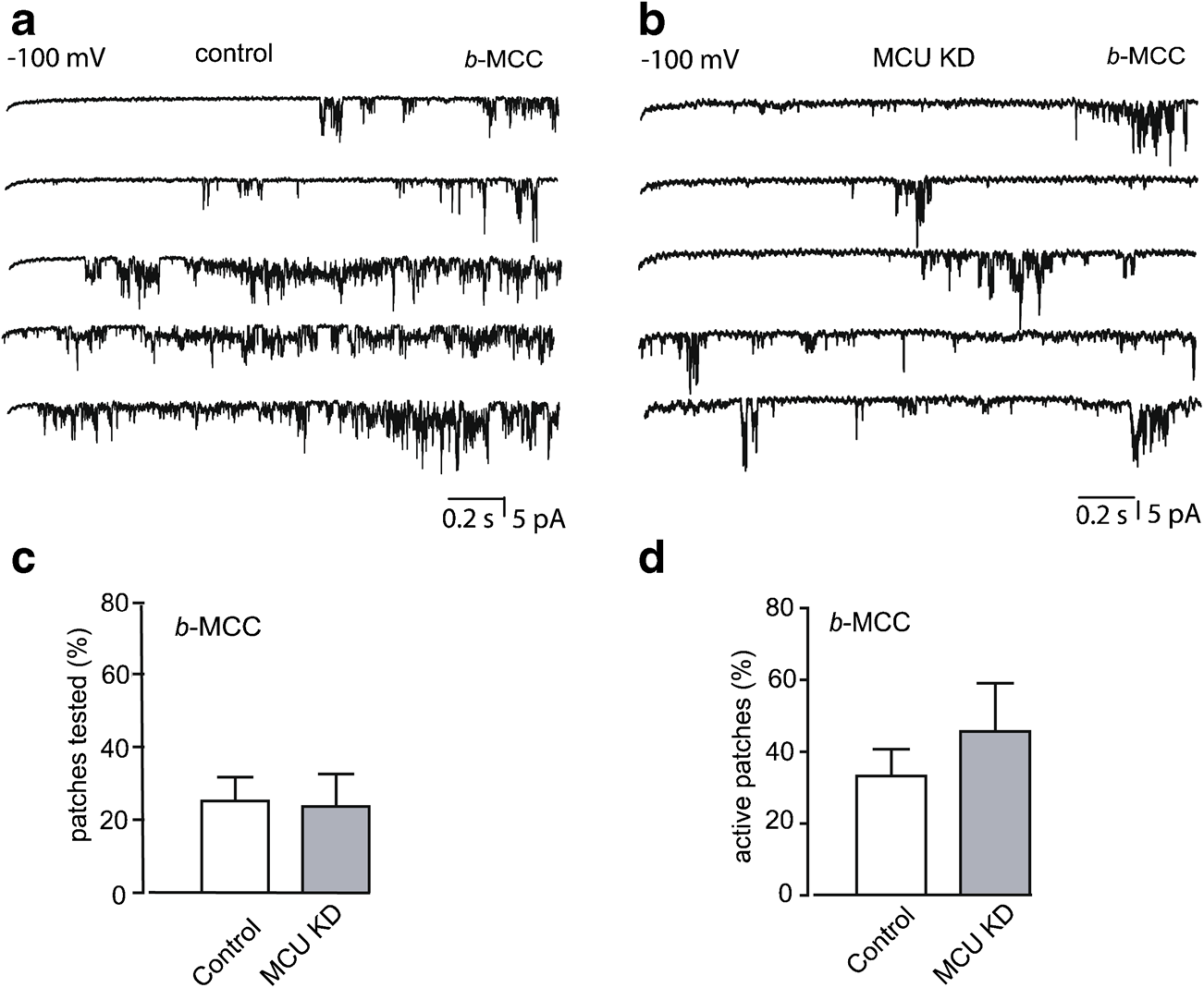
MCU knockdown does not affect the occurrence probability of *b*-MCC. **a** Representative single channel recording of *b*-MCC activity at a holding voltage −100 mV in mitoplast isolated from control cells. **b** Representative *b*-MCC recording from mitoplast isolated from MCU-KD HeLa cells. **c** *Bars* represent the occurrence of *b*-MCC activity in mitoplasts from control and MCU-KD HeLa cells in respect to the total number of patches. **d** The same as in **c** but in respect to the number of active patches with any MCC activity

Similar to mitoplasts isolated from control cells (Fig.5a), the second type of the channel activity studied in mitoplasts isolated from MCU-KD cells was the bursting activity (Fig.5b). According to our previous reports, we refer to this channel as bursting mitochondrial Ca^2+^ channel (*b*-MCC) [2]. Neither occurrence nor conductance of *b*-MCC was altered in mitochondria isolated from MCU-KD cells. In control cells, this channel was observed in 23 out of 103 patches tested. The probability of occurrence of this channel activity was 25 ± 6 % (*N* ^D^ = 32) in control group and 23 ± 9 % (*N* ^D^ = 13) in the MCU-KD group in respect to all patches tested (Fig.5c). Within mitoplasts from MCU-KD cells, the *b*-MCC activity was detected in 16 out of 67 patches tested (35 active patches) and the occurrence probability in respect to active patches was slightly higher (45 ± 13 %, *N* ^D^ = 11) compared to the control group (33 ± 7 %, *N* ^D^ = 29) (Fig.5d). In mitoplasts isolated from control cells, mean conductance of *b-*MCC channel was 25.7 ± 1.3 pS (*n* = 8), and in MCU-KD mitoplasts, the channel conductance was unaltered and averaged 26.0 ± 1.8 pS (*n* = 9) (Table 1).

### Stable knockdown of MCU increased the occurrence of the *xl-*MCC

**Fig. 6:**
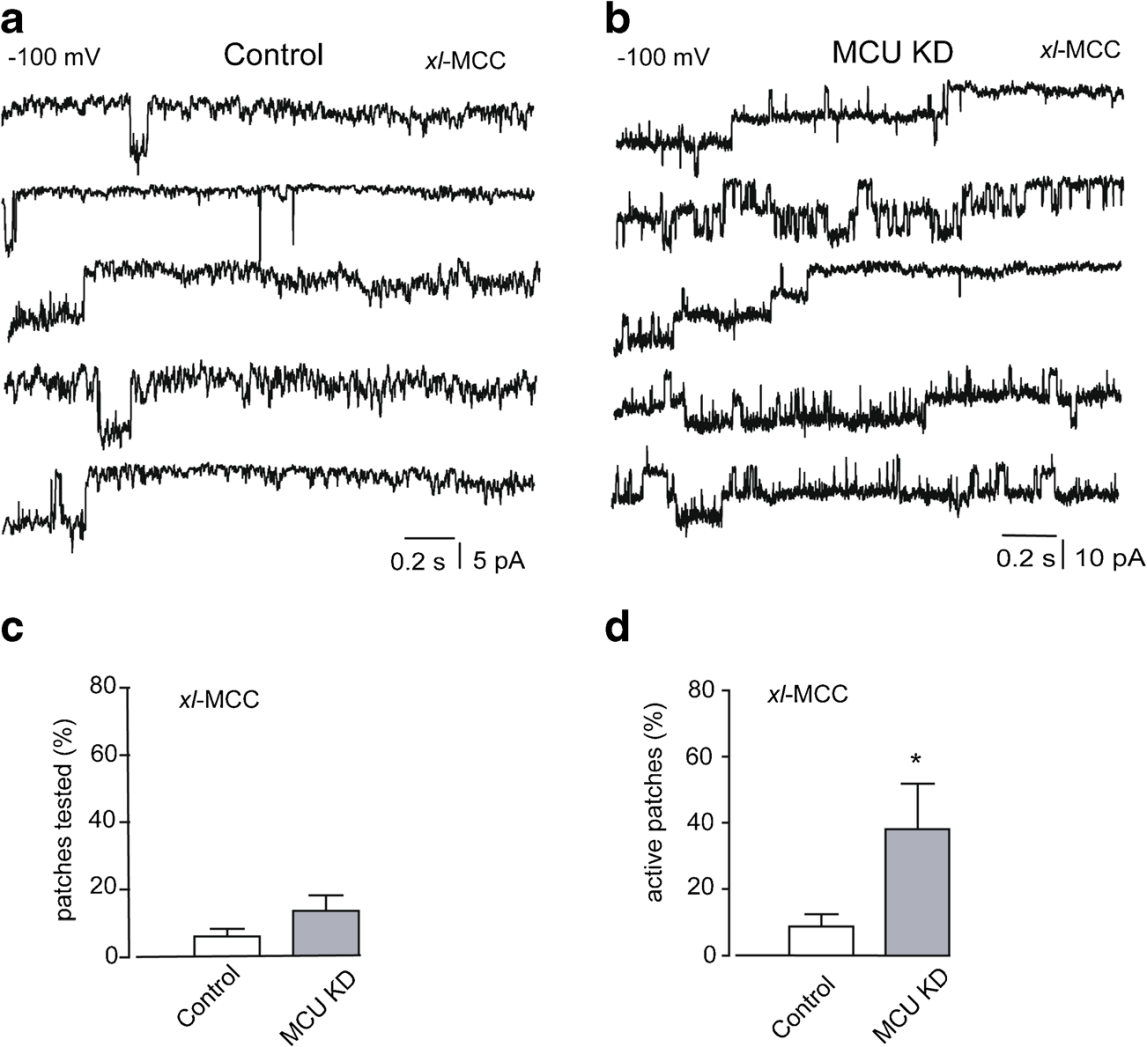
Effect of MCU knockdown on the occurrence of *xl*-MCC activity. **a** and **b** Representative single channel recording of *xl*-MCC activity at a holding voltage −100 mV in mitoplasts isolated from control (**a**) and MCU-KD cells (**b**). **c** *Bars* represent the occurrence of *xl*-MCC activity in mitoplasts from control and MCU-KD HeLa cells in respect to the total number of patches tested. **d** The same as in (**c**) but in respect to the number of active patches with any MCC activity

Both in control (Fig.6a) and MCU-KD group (Fig.6b), we also identified a third type of activity that we define as *xl*-MCC [2, 9]. In the control HeLa cells, *xl-*MCC conductance was 74.8 ± 7.9 pS (*n* = 8), thus, comparable to that previously reported [2]. Diminution of MCU did not affect significantly (*p* > 0.05) *xl*-MCC conductance 70.7 ± 5.9 pS (*n* = 6) (Table 1). Remarkably, in the control group, this type of activity was the least frequent and was observed in 8 out of 103 patches tested, while in MCU-KD mitoplasts, this type of channel activity was observed in 10 out of 67 patches tested. The occurrence probability of this channel in respect to all patches studied was 6 ± 2 % (*N* ^D^ = 32) in the control group and 13 ± 5 % (*N* ^D^ = 13) in MCU-KD group, indicating a 2.3-fold increase in the occurrence probability of *xl-*MCC in MCU-KD mitoplasts (Fig.6c). When compared with respect to the number of active patches, the occurrence probability of *xl-*MCC in the MCU-KD group showed 4.3-fold increase from 9 ± 4 % (*N* ^D^ = 29) in the controls to 38 ± 14 % (*N* ^D^ = 11) in the MCU-KD group (Fig.6d). Gating characteristics of *xl*-MCC were also affected by MCU knockdown and revealed a significant reduction in NPo, To_mean_, while Tc_mean_ was not significantly altered (Table 1).

## Discussion

MCU has recently been identified as the ion-conducting pore in the mitochondrial inner membrane [1, 4]. However, several other studies have pointed for alternative putative channels/carriers for mitochondrial Ca^2+^ influx including mitochondrial ryanodine receptors [20, 21], the Ca^2+^/H^+^ antiporter leucine zipper EF hand-containing transmembrane protein 1 [10, 11, 25], the uncoupling proteins 2 and 3 [22, 23, 26], and the canonical transient receptor potential 3 channel [6]. Moreover, so far, two regulator proteins for mitochondrial Ca^2+^ uptake, the MICU1 [15, 18] and the MCUR1 [14], have been described, thus supporting the concept of a multiprotein complex being responsible to establish the mitochondrial Ca^2+^ uniporter phenomenon [8, 19]. While single channel measurements of mitochondrial Ca^2+^ channels in the mitoplast-attached configuration recently confirmed the existence of multiple mitochondrial Ca^2+^ entry pathways [2, 9, 17, 21], the actual proteins that account for the individual channels are unknown. Therefore, in the present study, we explored the effect of MCU knockdown on the occurrence probability of distinct types of single channel activities in the inner mitochondria membrane of HeLa cells and the amplitude of whole-mitoplast inward Ca^2+^ and Na^+^ currents.

In whole-mitoplast configuration, diminution of MCU considerably reduced the inward Ca^2+^ current, an observation similar to that published previously [3]. Applying voltage ramps in divalent-free conditions produced a development of a linear inward current upon switching from Na^+^-free to Na^+^-containing solution. The current was sensitive to ruthenium red and had higher amplitude than the current developed when Ca^2+^ was added to the bath in the absence of Na^+^, indicating that in the absence of Ca^2+^, the channels permeate Na^+^, an observation consistent with the previous ones [7, 13]. We used this intrinsic property of mitochondrial Ca^2+^ permeable channel(s) to better discriminate the consequences of MCU silencing on electrical signaling of mitoplasts. Here, we show that MCU knockdown effectively suppresses transmitochondrial currents carried by Ca^2+^ and Na^+^. The degree of I_Ca_ and I_Na_ suppression upon MCU knockdown corresponded well to the degree of suppression of mitochondrial Ca^2+^ accumulation in intact cells upon histamine exposure. These findings confirm a very recent report that describes a large reduction of ruthenium red-sensitive whole-mitoplast currents of HEK293 cells with RNAi-mediated knockdown of the MCU [3].

In addition to the evaluation on the knockdown of MCU on ruthenium red-sensitive whole-mitoplast currents, we also explored whether MCU knockdown affects the occurrence probability of the individual and distinct single channel activities previously reported in HeLa mitoplast [2]. We found that the occurrence probability of active patches has been largely reduced in MCU-KD mitoplasts, thus supporting the concept of MCU being the main conducting pore of mitochondrial Ca^2+^ currents. However, we found that this reduction is mostly due to reduced occurrence probability of *i*-MCC channel that represents one (i.e., *i-*MCC; app. 14.3 pS) [9] out of three Ca^2+^ currents (*i-*MCC, *b-*MCC, *and xl-*MCC) in mitoplasts isolated from HeLa cells [2]. Although it was shown that purified MCU shows channel activity in lipid bilayers where under symmetrical 100 mM Ca^2+^ conditions the channel conductance was reported to be 6–7 pS [4], one can speculate that in its natural environment and under asymmetrical Ca^2+^ conditions, the MCU conductance may differ, possibly due to the formation of hetero-multimers. This assumption is in line with other reports on native mitoplasts isolated from cardiac myocytes and endothelial cells where two different channels with the conductance of app.13-14 and 7–8 pS have been discriminated under asymmetrical Ca^2+^ conditions [9, 17]. Accordingly, the selective decrease in occurrence probability of *i-*MCC upon MCU knockdown observed in the present study suggests that this type of activity is indicative for the MCU-established current.

The other observation of the present study is that MCU knockdown yielded an increased occurrence probability of *xl*-MCC channel activity in respect to active channels. This observation indicates that *xl-*MCC (app. 74–77 pS) [2, 9] is independent from the presence of MCU protein and mitochondrial Ca^2+^ channels other that MCU play a compensatory role under functional MCU diminution. Notably, our statistical analysis regarding the individual gating characteristics of *i-*MCC and *xl-*MCC revealed a decrease in the mean NPo and To_mean_ of both channels in mitoplasts from MCU-KD cells. However, in view of the rather large variances in these measurements, caution is necessary in the interpretation of these changes. Nevertheless, these data further support the concept of a rather complex mitochondrial Ca^2+^ uptake machinery that might consist from MCU-dependent and MCU-independent pathways that are functionally interrelated to meet the versatile Ca^2+^ demand of the organelle under different conditions of high and low metabolic and ion fluxes.

It is still unclear whether *b*-MCC and *xl-*MCC represent distinct or the same channel protein. However, because of observation that *b*-MCC could turn into *xl-*MCC activity, it is reasonable to suggest that a single channel pore protein accomplishes two distinct activities. Because the pipette solution for single channel recordings in the present study contained CGP37157, an inhibitor of NCX_mito_, which partially inhibits pH-dependent Ca^2+^ transport (Letm1), and because *xl-*MCC is a channel, mitochondrial Na^+^/Ca^2+^ exchanger(s) and Letm1 can be excluded from being responsible for *xl*-MCC current. Thus, further studies are needed to identify the molecular player(s) governing the *xl*-MCC activity.

Overall, the present study addressed the role of MCU in transmitochondrial Ca^2+^ fluxes using the direct patch-clamp approach on mitoplasts isolated from HeLa cells with diminished MCU expression and respective controls. Our current study shows that MCU knockdown results in a strong decrease in whole-mitoplast current and the number of active patches with Ca^2+^ channel behavior. Notably, this decrease is exclusively due to a decrease in the number of active recently identified *i*-MCC but not *b*-MCC or *xl*-MCC of which the latter one seems to play a compensatory role under conditions of MCU knockdown. Nevertheless, as gating characteristics for *i-*MCC and *xl-*MCC were affected by diminution of MCU, our findings point to a modulatory interaction between the two independent Ca^2+^ currents the nature of which awaits to be identified.
